# PCB-Based Magnetometer as a Platform for Quantification of Lateral-Flow Assays

**DOI:** 10.3390/s19245433

**Published:** 2019-12-10

**Authors:** Mohammad Khodadadi, Long Chang, João R. C. Trabuco, Binh V. Vu, Katerina Kourentzi, Richard C. Willson, Dmitri Litvinov

**Affiliations:** 1 Center for Integrated Bio & Nano Systems, University of Houston, Houston, TX 77204, USA; lvchang@central.uh.edu (L.C.); willson@uh.edu (R.C.W.); litvinov@uh.edu (D.L.); 2 Materials Science & Engineering, University of Houston, Houston, TX 77204, USA; 3 Department of Electrical & Computer Engineering, University of Houston, Houston, TX 77204, USA; 4 Department of Chemical and Biomolecular Engineering, University of Houston, Houston, TX 77204, USA; jrctrabuco@gmail.com (J.R.C.T.); vu.binh@gmail.com (B.V.V.); edkourentzi@uh.edu (K.K.); 5 iBB-Institute for Bioengineering and Biosciences, Department of Bioengineering, Instituto Superior Técnico, Universidade de Lisboa, 1049-001 Lisbon, Portugal; 6 Department of Biology and Biochemistry, University of Houston, Houston, TX 77204, USA; 7 Department of Chemistry, University of Houston, Houston, TX 77204, USA

**Keywords:** biosensor, sensor, magnetic

## Abstract

This work presents a proof-of-concept demonstration of a novel inductive transducer, the femtoMag, that can be integrated with a lateral-flow assay (LFA) to provide detection and quantification of molecular biomarkers. The femtoMag transducer is manufactured using a low-cost printed circuit board (PCB) technology and can be controlled by relatively inexpensive electronics. It allows rapid high-precision quantification of the number (or amount) of superparamagnetic nanoparticle reporters along the length of an LFA test strip. It has a detection limit of 10^−10^ emu, which is equivalent to detecting 4 ng of superparamagnetic iron oxide (Fe_3_O_4_) nanoparticles. The femtoMag was used to quantify the hCG pregnancy hormone by quantifying the number of 200 nm magnetic reporters (superparamagnetic Fe_3_O_4_ nanoparticles embedded into a polymer matrix) immuno-captured within the test line of the LFA strip. A sensitivity of 100 pg/mL has been demonstrated. Upon further design and control electronics improvements, the sensitivity is projected to be better than 10 pg/mL. Analysis suggests that an average of 10^9^ hCG molecules are needed to specifically bind 10^7^ nanoparticles in the test line. The ratio of the number of hCG molecules in the sample to the number of reporters in the test line increases monotonically from 20 to 500 as the hCG concentration increases from 0.1 ng/mL to 10 ng/mL. The low-cost easy-to-use femtoMag platform offers high-sensitivity/high-precision target analyte quantification and promises to bring state-of-the-art medical diagnostic tests to the point of care.

## 1. Introduction

The ever-growing role of molecular biomarkers in disease diagnosis and prognosis, as well as the prediction and assessment of treatment outcomes, calls for effective tools for clinically actionable biomarker detection and measurement. Many such tools capable of highly quantitative biomarker detection have been developed and are readily available in state-of-the-art centralized clinical laboratories. However, there remains a critical need for low-cost, easy-to-use, point-of-care diagnostic assays that can help facilitate healthcare in resource-limited environments and lead to improvements in diagnostics, treatment, and patient outcomes.

The lateral-flow assay (LFA) platform—the technology underlying the home pregnancy test—has been extensively explored for applications in rapid and inexpensive point-of-care medical diagnostics due to its low cost, ease of use, and sensitivity sufficient for many applications. A typical LFA readout is based on observing a color change of the test line due to the selective accumulation of reporters in the test line volume. Visual information is limited to the reporters near the surface of the test strip, with limited or no ability to capture the signal from the bulk of the test strip. Analyte quantification is challenging as a result of variations in lighting, sample volume, and the operator’s visual acuity. Despite numerous advancements in colorimetric labels, including gold nanocages [1], nanotubes [2], carbon nanoparticles [3], and cellulose nanobeads [4], among others, the sensitivity, signal range, and quantifiability of colorimetric label based LFAs remain a challenge. The use of other optical labels such as fluorescent dyes [5,6] or luminescent nanoparticles [7,8] faces various challenges due to reagent complexity, cost, and reader complexity [9].

In this study, an ultrahigh-sensitivity inductive transducer, the femtoMag, designed for the detection and quantification of superparamagnetic nanoparticle reporters immuno-captured in the volume of the test line of an LFA membrane, is demonstrated. Magnetic reporters provide a number of unique advantages over their optical counterparts: (1) magnetic fields do not interact with biological materials, so magnetic field-based detection is immune to signal degradation and distortion inherent to optical detection; (2) magnetic fields are not affected by LFA media, so every magnetic reporter within the test line volume contributes to detection; and (3) the properties of magnetic reporters can be tuned to match the biomarkers to optimize trapping efficiency and detection [10,11,12]. The femtoMag also provides a number of technological advancements over the current state-of-the-art magnetic biosensor technologies, including (1) volumetric detection, (2) high sensitivity, (3) direct quantitation, (4) easy integration with LFA technology, (5) portable electronic controls, and (6) low-cost manufacturability using conventional high-throughput PCB manufacturing technology. 

**Figure 1:**
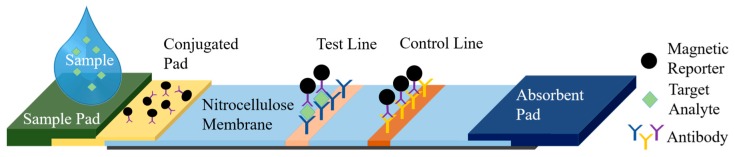
Lateral flow assay using magnetic nanoparticle reporters.

The femtoMag platform presented here enables LFA readout quantification using superparamagnetic nanoparticles as reporters. A typical LFA comprises a sample pad, a conjugate release pad, a test line, a control line, and an absorbent pad as illustrated in Figure 1. A sample containing the analyte of interest is applied to the sample pad. Capillary forces drive the sample through the nitrocellulose membrane towards the absorbent pad. As the sample flows through the conjugate pad, it interacts with and releases predeposited reporters, which are functionalized to bind to the target analyte. Superparamagnetic reporters will be captured by capture antibodies at the test line if the target analyte is present (sandwich assay). The control line is functionalized to capture magnetic reporters regardless of the presence of the target analyte. Any excess fluid is wicked by the absorbent pad. Magnetic reporters trapped in the test and control lines are detected by the femtoMag. The signal at the control line validates the test and the strength of the signal at the test line reflects the concentration of the targeted analyte. 

In this work, we demonstrate the quantitative measurement of superparamagnetic Adembead reporters trapped in the test line of an LFA test strip, using the femtoMag at room temperature. The femtoMag design achieves comparable sensitivities to the recently reported induction-based biosensor by exploiting the nonlinear magnetic behavior of magnetic nanoparticle reporters by exciting the reporters with a combination of two frequencies with magnetic fields strong enough to saturate the material [13,14].

With the exception of an exceedingly complex detection modality (not suitable for routine field applications) applied in conjunction with a conventional ac susceptometer [15], there are no published reports of magnetic nanoparticle inductive detection sensitivities approaching the femtoMag within 1000-fold. A benchtop susceptometer can detect ~60 billion 50 nm superparamagnetic Fe_3_O_4_ nanoparticles (20 μg) [16], while microfabricated planar coil sensors can detect 2 million 400 nm superparamagnetic Fe_3_O_4_ nanoparticles (0.3 μg) [17,18]. On the other hand, inductive nanofabricated transformers can detect the presence of a single 1 µm magnetic bead but require the bead to settle inside a 1 um ring, precisely [19,20].

The femtoMag platform may boost LFA sensitivity well in excess of what is practically achievable with ELISA (enzyme-linked immunosorbent assay), the gold standard for immunoassay diagnostics, or with any other technology of comparable cost (~$3 per a disposable chip cartridge and a ~$300 cartridge reader). Furthermore, compared to other point-of-care assays, our low-complexity LFA approach is expected to be among the easiest to use, far more sensitive than most (standard colorimetric LFA [21] and light scattering) and significantly less expensive than other assays of potentially sufficient sensitivity (upconverting phosphor LFAs [22], rotor/CD devices [23], immuno-PCR on emerging moderate-complexity PCR devices [24], or microfluidics [25,26]).

## 2. PCB-Based Magnetometer

**Figure 2:**
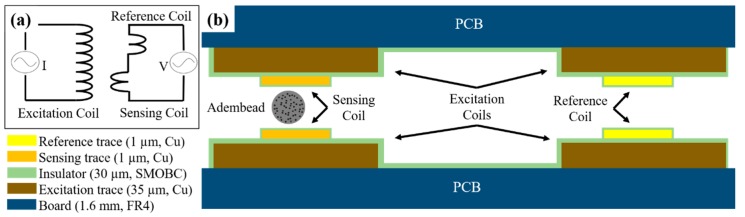
(**a**) The femtoMag uses an excitation coil to generate a magnetic field. The detector voltage is equal to the induced voltage at the sensing coil minus the induced voltage at the reference coil. (**b**) Schematic of the cross-sectional view of the femtoMag with a single Adembead reporter in the sensing coil.

The femtoMag is a differential inductive transducer designed to seamlessly integrate with an LFA. A schematic depicting the operating principles of the femtoMag is shown in Figure 2a. An excitation coil is used to generate an alternating magnetic field across the detector. The detector comprises a reference coil and a sensing coil connected in series but wound in opposite directions. In the absence of magnetic material in the sensing coil, the induced voltage across the detector is equal to zero. Magnetic materials such as superparamagnetic nanoparticle reporters in the sensing coil disrupt the balance, resulting in a non-zero readout voltage that is proportional to the amount of magnetic material in the sensing coil. This differential measurement minimizes environmental noises and enables precise voltage measurements on the order of 1 μV.

The femtoMag transducers used in this work are manufactured using conventional printed circuit board (PCB) technology. A low-cost ($5/in^2^ for three chips) and fast-turnaround time (seven days to ship) prototyping manufacturing service provider OSHPark was used [27]. Figure 2b is a schematic depicting the femtoMag transducer. The transducer is constructed using a pair of 2-layers PCB boards (1.6 mm thick FR4 substrate). The excitation coil is a copper trace on one side of a PCB. The copper trace is 10 mm long × 1 mm wide × 0.035 mm thick with an electroless nickel immersion gold (ENIG) finish. The excitation coil is insulated using solder mask over bare copper (SMOBC). This part is fabricated by OSHPark. The sensing coil and the reference coil is a microfabricated copper trace above the excitation coil. The microfabricated copper trace is 10 mm long × 0.25 mm wide × 0.001 mm thick and is deposited via shadow mask electron beam evaporation directly above the excitation coil. In future implementations, the femtoMag transducers will be manufactured using dual-layer PCB manufacturing technology to eliminate the need for shadow mask evaporation of the sensing and reference coils. The pair of PCBs are aligned using four pins that pass through the four alignment through-holes in the PCBs. The gap between the PCBs is set using precision spacers (stainless steel thickness gauge) before soldering the alignment pins to fix the assembly.

The sensing coil induced voltage, due to the presence of superparamagnetic nanoparticle reporters, can be determined by calculating the flux change generated by a magnetic dipole under the influence of an alternating magnetic field. The flux density generated by a magnetic dipole is given by [28,29]

(1) $$B=\frac{\mu_{0}}{4\pi }\left(\frac{3\left(m.r\right)r}{r^{5}}-\frac{m}{r^{3}}\right)=\frac{\mu_{0}m}{4\pi }\left(\frac{3xz}{r^{5}}\hat{i_{x}}+\frac{3yz}{r^{5}}\hat{i_{y}}+\frac{3z^{2}-r^{2}}{r^{5}}\hat{i_{z}}\right)=\frac{\mu_{0}V\chi H_{a}}{4\pi }\left(\frac{3xz}{r^{5}}\hat{i_{x}}+\frac{3yz}{r^{5}}\hat{i_{y}}+\frac{3z^{2}-r^{2}}{r^{5}}\hat{i_{z}}\right),$$

where χ is the susceptibility of the magnetic reporter; $H_{a}$ is the external magnetic field; V is the volume of the particle; $\mu_{0}$ is the permeability of free space; r is the radial vector from the center of the magnetic particle; and x, y, and z are the Cartesian distance from the center of the nanoparticle. 

From Faraday’s law, only the normal (z) component of the magnetic flux contributes to the induced voltage. The normal component of the magnetic flux density from a magnetic dipole located at the center of xy plane of a rectangular induction coil with dimensions of x=2t and y=2h and distance z=2p form the xy plane is

(2) $$\phi_{z}=\int_{-h}^{h}\int_{-t}^{t}B_{z}dxdy=\frac{\mu_{0}V\chi H_{a}}{\pi }\left(\frac{ht\left(t^{2}+h^{2}+2p^{2}\right)}{\left(h^{2}+p^{2}\right)\left(t^{2}+p^{2}\right)\sqrt{\left(t^{2}+h^{2}+p^{2}\right)}}\right)=\alpha V\chi H_{a},$$

where $\alpha =\frac{\mu_{0}V\chi H_{a}}{\pi }\left(\frac{ht\left(t^{2}+h^{2}+2p^{2}\right)}{\left(h^{2}+p^{2}\right)\left(t^{2}+p^{2}\right)\sqrt{\left(t^{2}+h^{2}+p^{2}\right)}}\right)$ is the induction coil shape factor. The time-varying flux results in an induced voltage given by [30]

(3) $$\varepsilon =-\frac{d\phi }{dt}=-\alpha fV\chi H_{a},$$

where f is the frequency of the excitation field. Equation (3) represents an approximate mathematical representation of the femtoMag readout voltage. The analytical model provides an intuitive understanding of the basic parameters that contribute to the induced voltage. To understand how the device might perform before committing resources to fabrication and experiments, we performed numerical simulations of the device using the boundary element magnetic field modeling package Amperes 3D by Integrated Engineering Software [31]. 

**Figure 3:**
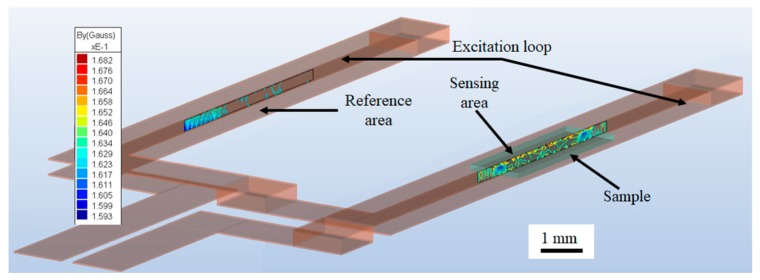
Amperes 3D is used to simulate the performance of the femtoMag design before fabricating the device for testing.

Simulating the femtoMag provides more accurate details on the magnetic field distribution in the sensing area and the reference area as shown in Figure 3. The red and blue regions represent the maximum and the minimum values of the magnetic field, respectively. The excitation coil is illustrated in transparent brown and the test line is outlined in transparent green. The magnetic reporters are assumed to be distributed uniformly within the test line and are modeled as a homogeneous magnetic material with a magnetic permeability corresponding to the number of trapped reporters. The difference between the flux in the sensing and reference area is used to calculate the induced voltage.

## 3. LFA Membrane and Test Sample Preparation

A model system based on the hCG pregnancy hormone was used to evaluate the performance of the femtoMag integrated with LFA nitrocellulose membranes. Whatman FF80HP nitrocellulose membranes (GE Healthcare) were used, with a length of 46 mm and a width of 3 mm. The membranes were functionalized with polyclonal anti-α hCG antibody (#ABACG-0500, Arista Biologicals, Allentown, PA, USA) at the test line and anti-mouse antibody (#ABGAM-0500, Arista Biologicals) at the control line. A BioDot XYZ3060 was used to dispense the antibodies (at 1 μg/cm) to form the test and control lines. Test samples were prepared using hCG model protein diluted in LFA buffer (1% Tween-20, 0.5% BSA, in PBS, pH 7.4), and 10 μL of each sample was mixed with 1 μL of 200 nm Adembead reporters functionalized with mouse monoclonal anti-β hCG antibodies (#ABBCG-0402, Arista Biologicals) [12]. Blank samples with no hCG were used as controls.

## 4. System Integration and Calibration

**Figure 4:**
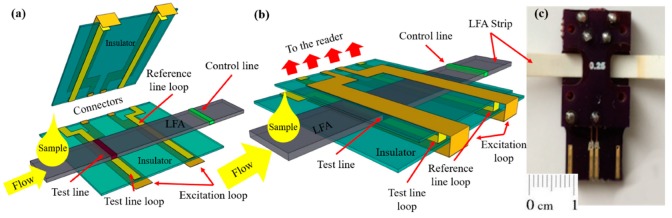
(**a**,**b**) A schematic of the proposed femtoMag biosensor (downstream absorbent pad not shown). (**c**) Image of the femtoMag.

The integration of the LFA membrane with the femtoMag transducer is illustrated in Figure 4. First, the LFA membrane is aligned with the bottom PCB board. The femtoMag chip has two identical detectors. While one of them acts as a sensing detector which is used to quantify the concentration of superparamagnetic reporters such as Adembeads in the test line, the second one act as a reference detector that is located away from the test line. Thus, the test line signal reflects the difference between the amount of Adembead reporters specifically trapped in the test line and the amount of Adembead reporters nonspecifically trapped in the LFA membrane. Figure 4c is a photograph of the prototype femtoMag chip. The gap on the chip is set at 0.3 mm to enable the 0.2 mm thick test strips to drag through. This allows a single detector on the femtoMag to measure both the test line and the control line by sliding the test strip through the transducer. The Adembead concentration along the strip was profiled as the LFA membrane was fed through the femtoMag transducer at a rate of 1 mm/s while taking one measurement every 10 ms.

The excitation coil of the femtoMag is driven by an RF generator, Rigol DG830, set to 5 Vp-p at 90 MHz. The spectrum analyzer—a Rigol DSA832E with an average noise level of 100 nV—was used to measure the induced voltage generated by the femtoMag. A linear actuator is used to drag a test sample through the femtoMag chip. A LabVIEW application was developed to control the data-acquisition.

## 5. Results and Discussion

The sample strips were prepared by dispensing 50 µL test samples with various hCG concentrations (from 0.1 to 10 ng/mL) onto the sample pad of each test strip. The sample strips were then washed with 200 μL LFA buffer dried at ambient conditions, and measured by dragging them through the sensor at a rate of 1 mm/s. Each sample strip was measured three times. Finally, we used a razor to extract the test line and control line to independently measure the amount of magnetic material using an alternating gradient field magnetometer (AGFM).

**Figure 5:**
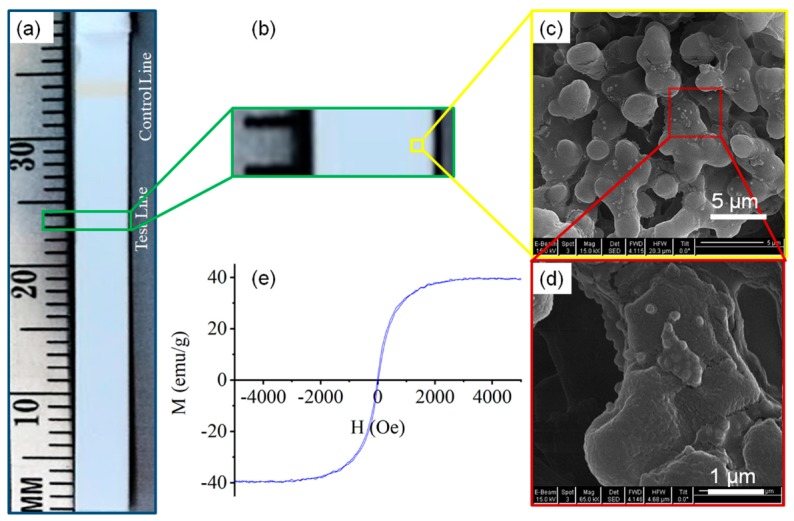
(**a**) A photograph of the 2.5 ng/mL LFA test strip (**b**) has a test line that is not visible to the naked eye, (**c**,**d**), but an SEM image reveals a minute amount of Adembead reporters on the test line. (**e**) Hysteresis loop of Adembeads measured by alternating gradient field magnetometer (AGFM).

An image of the 2.5 ng/mL hCG test strip (with test line not detectable by the naked eye) is shown in Figure 5a. A scanning electron image (SEM) of the test line revealing sparse loading with Adembead reporters on the nitrocellulose is shown in Figure 5c. The Adembead reporters (Carboxyl-Adembeads, #02122, Ademtech, Pessac, France) are monodisperse and superparamagnetic iron oxide (Fe_3_O_4_) nanoparticles encapsulated by a highly cross-linked hydrophilic polymer shell [32]. The M–H loop for the Adembead reporter was measured with an AGFM to determine the magnetic susceptibility of 0.17 (Figure 5e).

**Figure 6:**
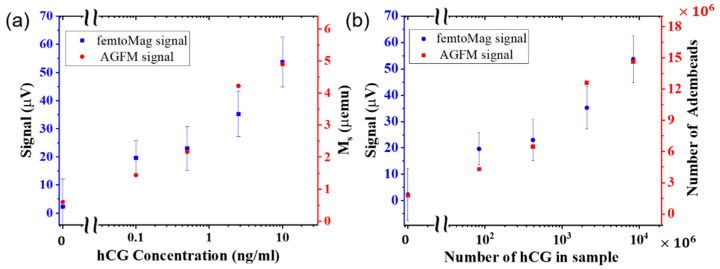
(**a**) The femtoMag (left axis) and AGFM (right axis) are used to quantify the number of magnetic nanoparticles immobilized in the test line of an LFA test strip loaded with various concentration of hCG pregnancy hormone. (**b**) The number of hCG molecules spent for each Adembead reporter in the test line.

The peak signal from the femtoMag is compared with the results of the AGFM in Figure 6a. The femtoMag peak signal and AGFM measurements show a close correlation that increases with hCG concentration. The signal for the control sample (hCG concentration of 0 ng/mL) is not zero because some of the Adembead reporters are trapped nonspecifically as they flow through the LFA membrane. Significantly, both the femtoMag and the AGFM can detect the minute amount of Adembead reporters nonspecifically trapped in the test line of the control sample. The nonspecifically bound reporters that may be trapped in the nitrocellulose membrane outside the lines is below the detection limit of both the femtoMag and AGFM. The femtoMag can reliably detect an hCG concentration below 100 pg/mL. The variability in the femtoMag signal (~12 µV) is approximately 12 times larger than the femtoMag noise floor (~1 µV), suggesting an opportunity to improve the sensitivity of detecting hCG to 0.01 ng/mL or below. 

From the AGFM measurements, the number of Adembead reporters trapped inside the test line is given by

(4) $$Number\;of\;Adembead\;=\;\frac{M_{T}}{M_{s}}\times \frac{1}{\rho V},$$

where $M_{T}$ is the magnetization saturation of the test line measured by AGFM, $M_{s}$ is magnetization saturation of the Adembead reporters (40 emu/g), ρ is the Adembead reporter density (2.0 g/cm^3^), and V is the Adembead reporter volume (4.2 × 10^−12^ mm^3^). A graph comparing the number of hCG molecules present in the test sample versus the number of Adembead reporters trapped in the test line is shown in Figure 6b. On average, this test requires approximately 100 hCG molecules for each Adembead reporter in the test line. 

**Figure 7:**
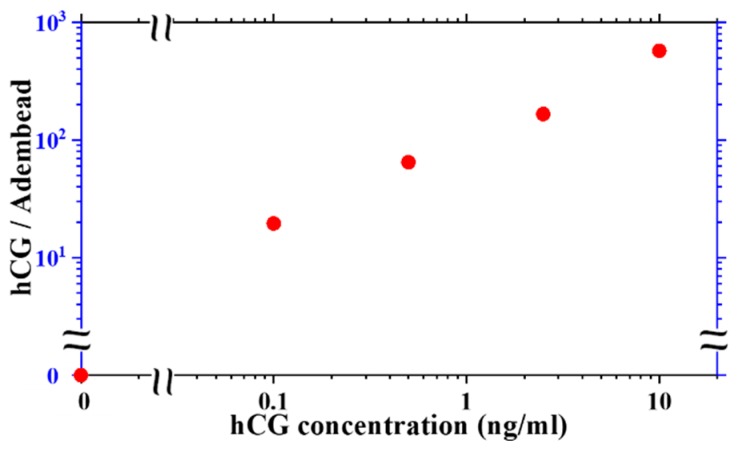
The ratio of the number of hCG molecules in the sample to the number of Adembead reporters measured in the test line increases monotonically with increasing hCG concentration.

The amount of hCG molecules in the sample per Adembead reporters found in the test line varies with hCG concentration as shown in Figure 7. The ratio of hCG molecules to the Adembead reporters in the test line increases monotonically from approximately 20 at a concentration of 0.1 ng/mL to 500 at 10 ng/mL. The hCG analyte is mixed with the Adembead reporters before applying them to the LFA, allowing its binding to the antibodies on the surface of the Adembeads to go to equilibrium. Therefore, the reaction kinetics of the reporters decorated with hCG as it flows through the available binding sites on the test line may be the limiting factor for the test sensitivity. Studying this phenomenon is beyond the scope of this report, but with the femtoMag, it is possible to methodically investigate this observation to understand the various factors (e.g., incubation time, the Adembead amount, and flow rate) that can improve the performance of the LFA.

**Figure 8:**
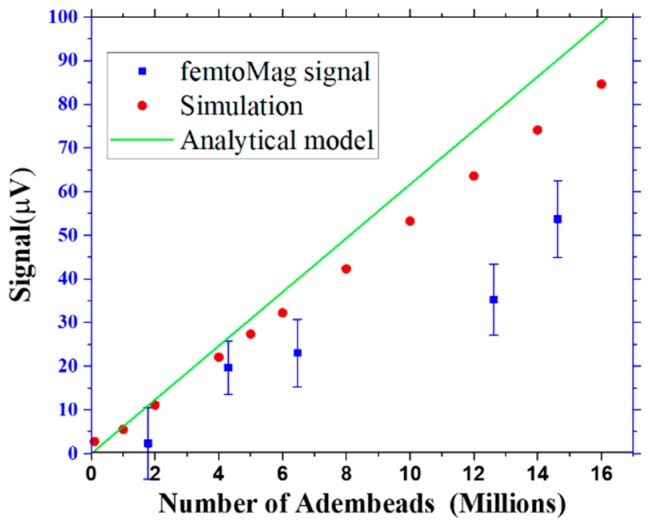
Comparison of the femtoMag signal from experimental measurements, analytical model and simulation results.

A comparison of the femtoMag signal to the predictions of the analytical model and the simulation is shown in Figure 8. The analytical model and simulation results approximately agree, and both overestimate the actual femtoMag signal, likely due to the overestimation of the excitation coil current. The femtoMag is driven by an ac voltage and the actual current flowing through the excitation coil depends on the coil’s impedance and the impedance of various components in the circuit. The femtoMag’s performance can be improved by optimizing the electronics to deliver greater power and by reducing parasitic resistances in the chip. Furthermore, since the femtoMag signal is linearly proportional to the drive current, the drive current can be used to increase the dynamic range of the measurement.

## 6. Conclusions and Future Work

The quantification of hCG in test samples using the femtoMag was demonstrated and verified using AGFM measurement. It was found that for every Adembead reporter found in the test line, there are approximately 100 hCG molecules in the sample. The ratio of target hCG molecules in the sample to the number of Adembead reporters measured at the test line increases monotonically from 20 to 500 as hCG concentration increases from 0.1 ng/mL to 10 ng/mL. The total number of Adembead reporters found in the test and control lines amounts to no more than 25% of the total number of Adembead reporters loaded into the sample. The femtoMag can make measurements every 1 ms as the sample strip is fed through the transducer to profile the number of magnetic reporters trapped along the LFA membrane. The ability to quickly and reliably quantify the number of magnetic reporters along the entire LFA strip provides a powerful tool to study the transport of magnetic reporters through an LFA membrane and their binding kinetics.

The femtoMag detector is an inductive sensor with a well-established principle of operation. An analytical model backed by simulations was used to design the current femtoMag transducer. There are many opportunities to improve LFA technology and the femtoMag can provide real-time, high throughput, and quantitative analysis of LFAs. Additional design optimization will help to further improve femtoMag sensitivity. Future work will also focus on improving the signal-to-noise ratio (SNR). SNR improvements are expected to help improve the limit of detection by at least a factor of 10 to enable the detection of 1,000,000 Adembead reporters.

In the current work, research-grade digital electronics were used to control the femtoMag. A linear actuator was also used to drag the sample strip through the femtoMag for measurements. A practical field-deployable biosensor can be developed with minor modifications to the system. There are many low-cost and small footprint options available to replace the waveform generator and voltmeter used in this work. It is possible that a femtoMag reader controlled and powered by a smartphone can be produced for less than $300. The femtoMag chip is currently manufactured at a cost of $3, which can be reduced by a factor of 10 when ordering 10,000 or more units. The assembly of the femtoMag with an LFA can readily be automated to improve quality, reliability, and throughput. For consumer use, the LFA will be fixed in the femtoMag assembly. Multiple detectors can be added without increasing the complexity of design or fabrication. The first detector quantifies the test line, the second detector validates the control line and a third detector can be used to quantify a second test line. Testing multiple target analytes (multiplexing) can help improve the overall sensitivity and specificity of the assay.

## 7. Patent

Litvinov, D.; Willson, R.C.; Chang, L.; Khodadadi, M. “Ultra-sensitive volumetric magnetic nanoparticle detector for quantifying lateral flow assays”, U.S. Provisional Pat. Ser. No.62798056. **2019**.
